# Prevalence of Hypomagnesemia among Elderly Patients attending a Tertiary Care Center: A Descriptive Cross-sectional Study

**DOI:** 10.31729/jnma.5764

**Published:** 2021-01-31

**Authors:** Surakchhya Gautam, Anju Khapung

**Affiliations:** 1 Department of Biochemistry, Kathmandu Medical College, Duwakot, Bhaktapur, Nepal; 2 Department of Community Dentistry, College of Dental Sciences, Nepal Medical College, Jorpati, Kathmandu, Nepal

**Keywords:** *elderly population*, *hypomagnesemia*, *serum magnesium*

## Abstract

**Introduction:**

Magnesium deficiency is common in the elderly and critically ill population and has been associated with a prolonged ICU stay. The knowledge of hypomagnesemia is essential as it could have prognostic and therapeutic implications in the elderly population. This study aimed to estimate the prevalence of hypomagnesemic in the elderly population visiting a tertiary care center.

**Methods:**

This descriptive cross-sectional study was conducted in a tertiary care hospital from March 21, 2020 to September 21, 2020. After obtaining ethical clearance from the institutional review committee (Ref. 2003202008), convenience sampling was done. Data were collected and entered in Microsoft Excel version 2007. Point estimate at 95% Confidence Interval was calculated along with frequency and proportion for binary data.

**Results:**

Out of 384 participants, 174 (45%) participants were found to have deranged magnesium levels, in which 111 (29%) (31.3-26.7 at 95% Confidence Interval) were found to be hypomagnesemia. Among them, 62 (29.4%) males and 49 (28.5%) females were hypomagnesemia. The average level of serum magnesium was 2.02±0.76 mg/dl ranging from 0.03 to 4.71. The mean age of participants was 70.31±8.13 years, among which the participants between the age group of 71-80 years presented with a maximum percentage of hypomagnesemia.

**Conclusions:**

The present study has shown that an apparently-healthy elderly population may have a magnesium deficiency that may need to be identified and treated for optimizing clinical care. Further multicentric studies with a greater sample size should be done in this field, which will benefit the elderly population.

## INTRODUCTION

Magnesium is the second most common intracellular cation after potassium. It is highly controlled with a normal range of 1.8-2.6 mg/dl.^1^ Its deficiency has been associated with several clinical manifestations such as arrhythmia, cardiac insufficiency, muscle weakness, and electrolyte imbalance.^2,3,4^ It can also lead to increased intracellular sodium and calcium concentration and increased peripheral resistance and vasospasm.^5^

Hypermagnesemia is rare, but hypomagnesemia, on the other hand, is more apparent in geriatric patients.^2^ The probable association of hypomagnesemia with geriatric patients is due to low intake, diminished intestinal absorption, increased urinary output, and different drug interactions.^3,6^

Clinically more attention is given to the electrolyte imbalance, but magnesium deficit is overlooked. But different studies have shown the importance of assessing magnesium levels in critically ill patients.^7^ The objective of this study was to find the prevalence of hypomagnesemia among the elderly population.

## METHODS

A descriptive cross-sectional study was conducted among patients attending Kathmandu Medical College and Teaching Hospital (KMCTH) at Sinamangal and Duwakot, Kathmandu, Nepal, from March 21, 2020 to September 21, 2020. The ethical approval was received from the Institutional Review Committee of KMCTH (ref 2003202008). The study population for this study comprised of OPD patients of age group 60 years and above. Population below 60 years and critically ill patients were not included in this study. Convenience sampling was done, and the sample size was calculated using the following formula,

$$\begin{array}{ll} \text{n} & ={\text{Z}}^{\text{2}}\times \text{p(}1-\text{p)}/{\text{e}}^{\text{2}}\\ & ={\left(1.96\right)}^{2}\times (0.5)\times (1-0.5)/{\left(0.05\right)}^{\text{2}}\\ & =384 \end{array}$$

where,

n = sample size
Z = 1.96 at 95% Confidence Interval (CI)
p = population proportion, 50%
e = margin of error, 5%

Written informed consent was obtained from all the study participants, and the data collection procedure was then carried out. Demographic data, including age and gender, were obtained on the proforma, and five ml of participant's blood was collected to examine the magnesium concentration in serum. Under the aseptic condition, a blood sample was obtained by venipuncture from the median cubital vein in a capped tube. Analysis of serum Magnesium was carried out using Xylidyl Blue colorimetric method utilizing Humalyzer Primus Version 3.2e semi auto analyzer in KMCTH laboratory, Duwakot.

Data were entered in Microsoft excel 2007 version and transferred to Microsoft Excel Sheet for the required analysis. Point estimate at 95% Confidence Interval was calculated along with frequency and proportion for binary data. We counted the number of patients with hypomagnesemia (serum magnesium ≤1.8mg/dl). We classified them as mild (1.4-1.8mg/dl), moderate (1.0-1.39mg/dl), or severe (<1.0mg/dl) and compared within the groups.^2^

## RESULTS

Out of the 383 study participants, 174 (45%) participants were found to have deranged magnesium levels, in which 111 (29%) at 95% CI [31.3-26.7] were found to be hypomagnesemic.

Among the total study participants, 211 (55.1%) were males, and 172 (44.9%) were females. The mean age of the study participants was 70.31±8.13 years. According to their age, the participants were categorized into three groups, 60-70 years, 70-80 years, and above 80 years. The majority of the study participants, 205 (53.5%) were between 60-70 years while 129 (33.7%) were between 70-80 years and 49 (12.8%) were above 80 years. In the current study, the average serum magnesium level (mg/dl) was 2.02±0.76 ranging from 0.03 to 4.71.

The prevalence of hypomagnesemia among the study participants was 111 (29%). The prevalence of hypomagnesemia was higher in males 60 (29.4%) than in females 49 (28.5%). Among the study participants of different age groups, those of 81 years and above had the highest prevalence of hypomagnesemia 26 (53.1%), as shown in Table 1.

**Table 1:** Distribution of study participants according to the prevalence of hypomagnesemia.

Variables		Hypomagnesemia n (%)
Sex	Male (n= 211)	62 (29.4)
	Female (n= 172)	49 (28.5)
Age group (years)	60-70 (n= 205)	48 (23.4)
	71-80 (n= 129)	37 (28.7)
	81 and above (n= 49)	26 (53.1)
Total (n = 383)		111 (29)

**Figure 1:**
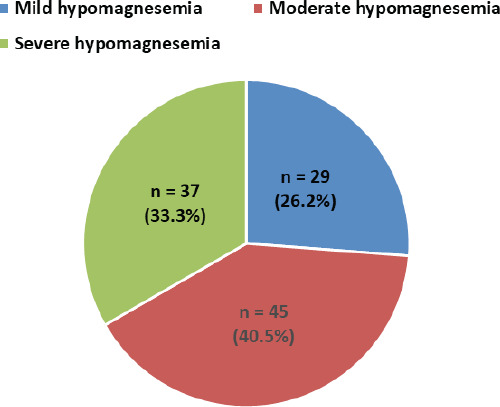
Distribution of hypomagnesemics according to the severity of hypomagnesemia

Among the hypomagnesemic study participants, a majority had moderate hypomagnesemia (40.5%) (Figure 1).

**Figure 2:**
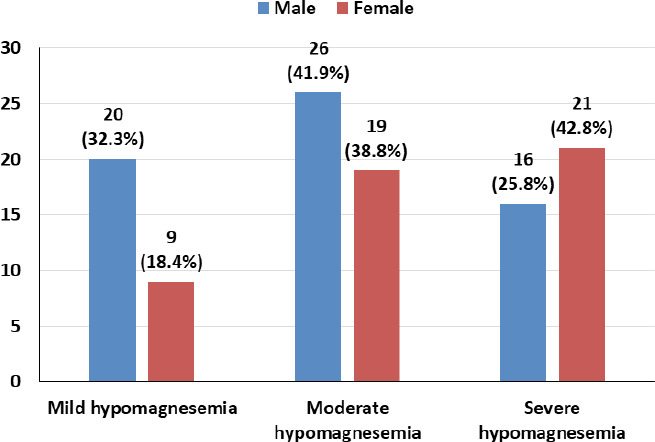
Distribution of male and female hypomagnesemics according to the severity of hypomagnesemia

The majority of the males (41.9%) had moderate hypomagnesemia, whereas most females (42.8%) presented with severe hypomagnesemia (Figure 2).

**Figure 3:**
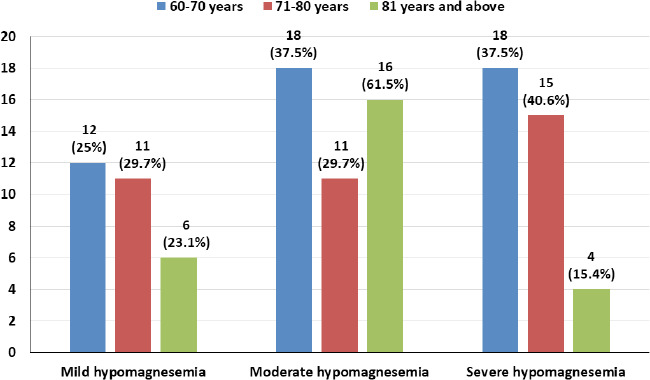
Distribution of different age groups of hypomagnesemics according to the severity of hypomagnesemia

Among the 60-70 years age group, the majority had moderate and severe hypomagnesemia 18 (37.5% each), and in the 71-80 years age group, the majority had severe hypomagnesemia 15 (40.6%). The prevalence of moderate hypomagnesemia was highest among the 81 years and above age group 16 (61.5%) (Figure 3).

## DISCUSSION

The senior citizens’ act 2063, Nepal defines senior citizens as people who are 60 years and above. Globally, the population of the elderly population is showing an increasing trend at 13% of the total population as of 2017.^8^ With a wide spectrum of association of serum magnesium levels in bodily function, the prevalence of hypomagnesemia, particularly in the geriatric population, needs a better understanding in the context of optimizing geriatric health care needs if a strong clinical association can be established. In this study, 62 (29.4%) males and 49 (28.5%) females had low serum Mg levels. Among hypomagnesemic male participants, 26 (41.9%) had moderate hypomagnesemia, and 16 (25.8%) had severe hypomagnesemia. Similarly, among the female hypomagnesemic participants,^1,9^ (38.8%) presented with moderate hypomagnesemia, while 21 (42.8%) had severe hypomagnesemia. While the prevalence of hypomagnesemia appeared to be greater in males, the severity of hypomagnesemia was found to be greater in female participants.

Similar to our findings, Lars et al. reported that enhanced Mg retention and decreased basal urinary output among the elderly compared to younger patients and Mg supplementation improved Mg status, suggesting that subclinical Mg deficiency is common in apparently healthy elderly patients.^9^ A study done in Nepal by Bijaya et al. also found that the mean serum value of Mg was less in patients receiving diuretics and Digitalis, which are commonly prescribed drugs among elderly patients.^5^ Huang et al. have also suggested that the majority of elderly type 2 diabetic patients may have low Mg intake and hypomagnesemia which was associated with metabolic abnormalities and depression. It also highlighted the need for regular serum Mg assessment on elderly, comorbid patients.^10^

A study done by Kumar et al. on patients above 60 years and admitted in ICU found that hypomagnesemia was associated with a slightly higher mortality rate. The requirement and duration of ventilator support were also higher, indicating that monitoring of serum Mg levels may have prognostic implications in the elderly.^3^ Similar study done by Martin et al. also found 10.7% of the total population (1576) had a serum magnesium level below 0.7mmol/l, with 4.4% having a serum level less than 0.65mmol/l.^11^ Davidovic et al. have suggested that Mg therapy can be performed within supplementation, and those doses are completely safe. In geriatric patients, the main importance is Mg's cytoprotective role, which could theoretically extend years of living.^6^ But, in Nepal, such studies are rare if not absent, as suggested by our literature search, highlighting the need for more research in this area.

In our study, in the age group 71-80 years, 37 (28.7%) were hypomagnesemic. Similarly, in the 81 and above age group, 26 (53%) were hypomagnesemic. This finding is greater than that of the cohort study done by Nasser et al., where they have taken 75 years and above study population, among which 6% had hypomagnesemia. The study has also reported that hypomagnesemia was associated with a higher 30-day mortality rate (18.4%) compared to normomagnesemic group (14.8%).^4^ In a study done by Arinzone, et al., hypomagnesemia was found in 36% of the patients, of whom 35% presented with moderate HM (0.8-0.9 microequiv./l) and 18% with severe HM (< or=0.7 microequiv./l). In their study, the sample population is above 65 years, like ours.^12^ Dandinavar et al., in their study, have concluded that child patients with hypomagnesemia had an increased duration of PICU stay and a higher mortality rate.^13^ Mahmond et al. concluded that children with attention deficit hyperactivity disorder had a lower level of serum Mg, which further focuses on assessing serum Mg level in children.^14^

Among the hypomagnesemic population in the age group 71-80 years, 15 (40.6%) had severe hypomagnesemia, while in the 81 and above age group, 16 (61.55%) had moderate hypomagnesemia, and 4 (15.4%) had severe hypomagnesemia. This finding indicates a higher prevalence of severe hypomagnesemia with increasing age. Similar findings were reported in a similar study done by Pokharel et al.15 The overall result of deranged magnesium levels in a total of 174 (44.9%) study participants itself is suggestive of the need for further exploration of this observation, which may positively influence geriatric health parameters by optimizing clinical care.

The study's limitation was that it is a single institution study, and the sample size isn't effective in formulating a consensus. The inclusion of critically ill patients could also have helped to broaden the understanding of the importance of hypomagnesemia in critical care settings.

## CONCLUSIONS

The present study has shown that an apparently-healthy elderly population may have Magnesium deficiency that may need to be identified and treated, optimizing clinical care. Although the prevalence of hypomagnesemia is high among males, the female population presented with a higher prevalence of severe hypomagnesemia. The higher prevalence of severe hypomagnesemia in the 71-80 years age group is an important finding that needs further exploration and clinical judgment while managing this population. Further multicentric studies with a greater sample size should be done in this field, which will benefit the elderly population.
